# Impact of chronic cold stress on metabolism, colostrum quality, and lamb performance in locally adapted ewes

**DOI:** 10.1007/s11250-026-04962-6

**Published:** 2026-03-05

**Authors:** Ayşe Uysal, Emre Yılmaz, Cihan Gür, Soner Uysal, Doğan Türkyılmaz, Ülkü Dağdelen Türkyılmaz

**Affiliations:** 1 Department of Animal Science, Faculty of Veterinary Medicine, Atatürk University, Erzurum, 25240 Türkiye; 2 Department of Animal Nutrition and Nutritional Diseases, Faculty of Veterinary Medicine, Atatürk University, Erzurum, 25240 Türkiye; 3 Department of Medical Services and Technologies, Vocational School of Health Services, Atatürk University, Erzurum, 25240 Türkiye; 4 Department of Animal Science, Faculty of Agriculture, Atatürk University, Erzurum, 25240 Türkiye

**Keywords:** Cold stress, Colostrum, Ewe, Lamb, Metabolic profile

## Abstract

This study evaluated the effects of chronic cold stress on metabolism, colostrum quality, and early lamb performance in two environmentally resilient sheep breeds: Morkaraman and Awassi. The study included 20 ewes (10 each breed) and their lambs (10 per breed). This study evaluated serum biochemical parameters and colostrum quality and composition in sheep and assessed the growth performance of lambs during the first seven days of life under cold environmental conditions. Findings indicated that Morkaraman lambs exhibited higher birth weights and superior growth rates compared to Awassi lambs under cold conditions. Cold stress significantly influenced serum biochemical parameters, with Morkaraman ewes showing higher triglyceride and gamma-glutamyl transferase (GGT) levels. Colostrum quality declined over time in both breeds; however, immunoglobulin G and GGT concentrations decreased more rapidly in Morkaraman ewes. In conclusion, Morkaraman sheep demonstrated better lamb growth performance under chronic cold stress, but this advantage was accompanied by a faster deterioration in colostrum quality, indicating breed-specific physiological responses to cold environments.

## Introduction

In traditional sheep farming, ewes’ gestation period usually falls during the winter, which in some parts of the world may expose ewes and their offspring to low ambient temperatures. In order to preserve homeostasis, animals normally react to cold stress by starting particular metabolic processes. The Hypothalamic-Pituitary-Adrenal (HPA) axis is activated during this process, increasing cortisol secretion to support energy balance and mobilize energy substrates. However, because of the influence of pregnancy-associated hormones and the need to shield the fetus from high cortisol levels, the HPA axis is not as activated during pregnancy in ewes. Pregnant ewes may be more vulnerable to negative effects in cold environments due to the decreased activation of the HPA axis (Verbeek et al. 2012; Verbeek et al. 2019). Sheep, like other ruminants, expend more energy in cold weather, which makes them more dependent on fats for energy (Zhang et al. 2022). In addition to the physiological challenges faced by ewes, cold stress poses a substantial risk to newborn lambs, particularly during the early postnatal period (Shi et al. 2022).

Cold, windy weather have been found to be major causes of neonatal lamb mortality; hypothermia brought on by these factors is responsible for almost half of perinatal lamb deaths (Tüfekci and Sejion 2023). The hances of newborn lambs surviving in the extrauterine environment prior to weaning are greatly increased when they consume high-quality colostrum (Farooq et al. 2024). Lamb health depends on a variety of bioactive substances found in colostrum, including proteins, fats, vitamins, minerals, lactose, hormones, enzymes, and peptides. These biofactors have growth-promoting, antimicrobial, and anti-inflammatory qualities that aid in the digestive system’s development. Furthermore, by supplying energy, colostrum supports energy homeostasis, especially in lambs born in harsh weather conditions (Agenbag et al. 2021). Colostrum’s beneficial effects on the health and survival of newborn lambs depend on its high quality. Breed, age, nutrition, dystocia, parity, and environmental stressors are some of the factors that affect the quality of colostrum (Uysal et al. 2024). Among these factors, breed-related differences in physiological adaptation to environmental stressors may play a particularly critical role in determining colostrum quality and neonatal resilience under cold conditions. Morkaraman and Awassi sheep breeds are characterized by their robust body conformation and strong adaptive capacity to harsh and variable environmental conditions, making them widely distributed in Türkiye and the Middle East, respectively (Şahin and Kopuzlu 2022; Ceyhan and Kozaklı 2023). Despite their widespread use in harsh climatic regions, comparative data on their physiological and productive responses to prolonged cold stress still remain limited. Thus, this study compares the metabolic profiles of ewes, the properties of colostrum, and the growth and health of newborn lambs in two sheep breeds that were subjected to cold stress during the lambing season.

## Materials and methods

### Ethical approval

The Atatürk University Animal Ethics Committee examined and approved the experimental protocols (Date: 30.03.2023, No: 2023–04–75).

### Study location and climate data

**Fig. 1:**
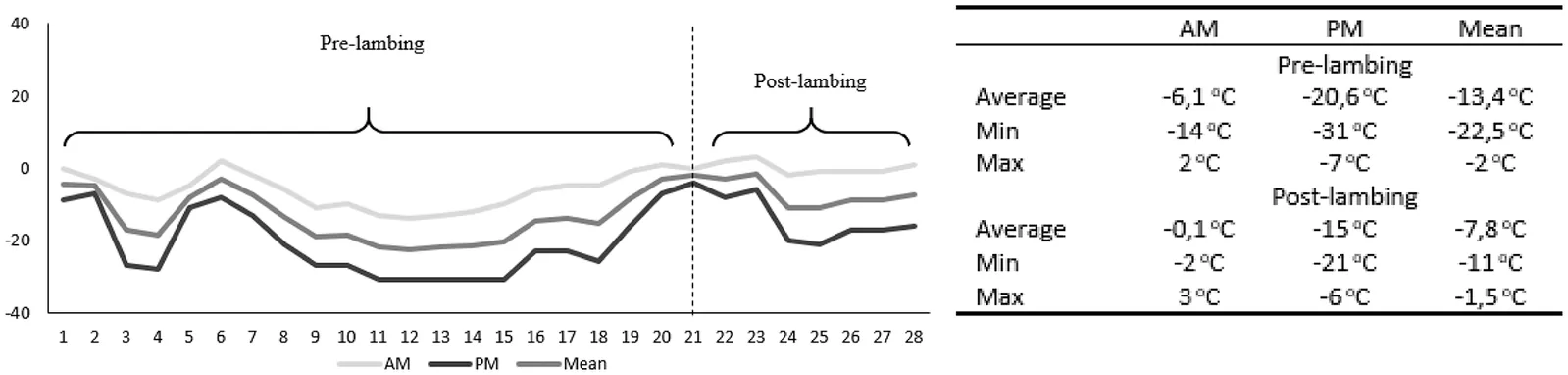
Mean daily ambient temperature values recorded throughout the experimental period. AM: Ante meridiem, PM: Post meridiem

In northeastern Türkiye (39°91’N, 41°22’E), at the Sheep Unit of the Atatürk University Food and Livestock Application and Research Center, the study was carried out from February to March 2023. Inside the shelter, a data logger (Cem, DT-38 F) was set up to continually record the outside temperature. Figure 1 presents the mean daily ambient temperature values recorded throughout the experimental period, from three weeks prior to the estimated lambing dates until one week postpartum.

### Feeds, animals, and study design

The study comprised 20 ewes (10 Morkaraman and 10 Awassi) that gave birth to a single lamb, together with their respective offspring (20 lambs). Although the sample size was limited, it was considered sufficient to provide preliminary comparative data under controlled cold-climate conditions. The experiment was conducted on a farm located in a cold-climate region, where the ewes were housed in semi-open shelters without additional heating throughout the gestation period (animals were naturally exposed to cold stress during the final trimester of pregnancy). To minimize potential age-related confounding effects, all ewes were carefully selected to be of similar age, to have at least one previous lambing history, and to be clinically healthy based on thorough veterinary examinations. Following lambing, the ewes were fed the diet formulated as presented in Table 1. The basal diet was analyzed for dry matter, crude ash, crude fiber, crude protein, crude fat, nitrogen-free extract, acid detergent fiber (ADF), and neutral detergent fiber (NDF) using standard procedures described by AOAC (1990) and Van Soest et al. (1991).

**Table 1:** Ingredients and nutrient composition of the basal diet of ewes

Ingredients	Amount, kg
Dry meadow hay	1.05
Concentre	0.650
Premix	0.050

Nutritional components	%
**Dry matter**	87.00
**Crude protein**	11.73
**Crude fiber**	19.00
**ADF**	21.50
**NDF**	37.50
**Ether extract**	3.34
**Ash**	9.00
**Nitrogen-Free Extract**	56.93
**Metabolic Energy** ^*^	9.80
**Calcium**	1.12
**Phosphorus**	0.74

* MJ/kg. Per animal; Vitamin A: 2500 IU, Vitamin D: 250 IU, Vitamin E: 50 mg, Vitamin K: 1 mg, Thiamine: 0.3 mg, Riboflavin: 0.5 mg, Pyridoxine: 0.5 mg, Cobalamin: 0.2 µg, Magnesium: 0.3 g, Sodium: 0.5 g, Chloride: 0.3 g, Selenium: 0.1 mg, Zinc: 40 mg, Manganese: 25 mg, Iron: 50 mg, Copper: 10 mg. The value was calculated and converted (Jurgens 1996)

### Sample collection

The first colostrum samples were collected within the first hour following parturition, and subsequent colostrum/milk samples were obtained at 12, 24, 36, 48, 60, and 168 h postpartum, resulting in a total of seven sampling points per ewe. Previous studies have demonstrated that the quality of colostrum progressively declines during the first three days after birth, transitioning into transitional milk and then mature milk by around the seventh day of lactation. In the present study, therefore, colostrum samples were collected at 12-hour intervals during the first three days postpartum, with an additional sample obtained on the seventh day of lactation (Abdel-Salam et al. 2019; Uysal et al. 2024). Animal caregivers kept a close eye on the births, and roughly 50 mL of colostrum/milk was taken in equal amounts from both udder lobes and placed in sterile tubes. Prior to analysis, all colostrum/milk samples were kept at -20 °C in sterile tubes. Blood samples were collected from lambs at 36 h after birth and from ewes at 2 h postpartum. The obtained serum samples were separated by centrifugation at 4000 rpm for 10 min and kept at − 20 °C until biochemical analyses.

### Determination of performance and rectal temperature of lamb

Birth weights within the first 24 h and on day 7 postpartum were recorded to assess early growth performance of lambs. Average daily weight gain was calculated based on the difference between consecutive body weight measurements., the rectal temperatures of lambs were immediately measured with a conventional thermometer after birth.

### Determination of colostrum quality

A Brix refractometer (Milwaukee Instruments, USA) was used to measure the density of colostrum at 1, 12, 24, 36, 48, 60, and 168 h after parturition. For the measurement of total solids (Brix), sample readings and calibration procedures were conducted in accordance with the manufacturer’s instructions. A few droplets of colostrum were placed on the prism of the refractometer, and then digital measurement was initiated by pressing the start button. After each measurement, the refractometer was washed with tap water, dried with a paper towel, and prepared for the following sample. The instrument was calibrated using distilled water at the beginning of each measurement period and after a maximum of 10 samples. The milk composition analyzer (Lactoscan Instruments, Bulgaria) was used to determine the nutritional composition of colostrum, including its fat-free dry matter, density, protein, ash, and pH.

### Determination of IgG, GGT, LDH, and ALP in blood serum and colostrum

The certain component concentrations in blood serum and colostrum was used to measure the Enzyme-Linked Immunosorbent Assay (ELISA) method. Sheep-specific ELISA kits from a commercial supplier (Sunlong Biotech, China) were used for the measurements. The IgG and GGT levels were assessed in colostrum, and IgG levels were measured in lamb blood serum. Non-esterified Fatty Acids (NEFA), Beta-Hydroxybutyrate (BHBA), and Blood Urea Nitrogen (BUN) levels were examined in blood serum of ewe and lamb. Before analysis, serum and colostrum samples that had been stored at -20 °C were first thawed at + 4 °C and then allowed to acclimate to room temperature. According the manufacturer’s protocol, the samples were appropriately diluted before the assay was conducted, which included the conjugate addition, washing stages, substrate reaction, and stopping solution. A microplate ELISA reader (Bio-Tek, USA) was used to record absorbance readings at 450 nm. To determine the component concentrations in the serum and colostrum samples, a standard curve was generated using the reference standards (Vetter et al. 2013).

### Analysis of other biochemical parameters in serum of ewe and lamb

The levels of total protein, albumin, globulin, total cholesterol, triglycerides, high density lipoprotein (HDL), low density lipoprotein (LDL), glucose, and GGT in ewe serum as well as total protein, albumin, globulin, and glucose in lamb serum was used to measure an AU500 automated biochemical analyzer (Beckman Coulter Inc., USA).

### Statistical analysis

The statistical program SPSS 20.0 was used to analyze the data collected for the study. Prior to statistical analyses, normality and homogeneity of variances were assessed using Shapiro–Wilk and Levene’s tests, respectively. Independent sample t-tests were used to determine breed differences for parameters evaluated at a particular time point after normality assumptions were confirmed (birth weight, body weight measurements, and blood biochemical parameters). A General Linear Model with the following structure was used for time-dependent variables (colostrum/milk composition). Breed, time, and the breed × time interaction were all fixed effects in the model. Polynomial contrasts were used to analyze linear and quadratic trends over time.

$${\mathrm{Y}}_{\mathrm{i}\mathrm{j}}=+{\mathrm{B}\mathrm{r}\mathrm{e}\mathrm{e}\mathrm{d}}_{\mathrm{i}}+{\mathrm{T}\mathrm{i}\mathrm{m}\mathrm{e}}_{\mathrm{j}}+{(\mathrm{B}\mathrm{r}\mathrm{e}\mathrm{e}\mathrm{d}\times\mathrm{T}\mathrm{i}\mathrm{m}\mathrm{e})}_{\mathrm{i}\mathrm{j}}+{\varepsilon}_{\mathrm{i}\mathrm{j}}$$

Where:

Y_ij_ = observed response variable for breed i at time j

µ = overall mean

Breed_i_ = fixed effect of breed (i = Awassi, Morkaraman)

Time_j_ = fixed effect of time (j = 1, 12, 24, 48,60,and 168 h post-partum)

(Breed × Time)_ij_ = interaction between breed and time

ε_ij_ = residual error

Pearson correlation coefficients were calculated to determine the correlations between factors that were normally distributed. All data were expressed as arithmetic means ± standard error of the mean. The threshold for statistical significance was set at *p* < 0.05.

## Results

### Growth performance and rectal temperature in lamb

No significant breed-related differences were observed in the birth weights and daily weight gains of the lambs (Table 2). The birth weight of Morkaraman lambs was numerically higher than that of Awassi lambs (*p* = 0.053). The study revealed a significant difference in rectal temperature values between Awassi and Morkaraman lambs within the first hour after birth. The rectal temperatures of Awassi lambs within the first hour after birth were significantly higher than those of Morkaraman lambs (*p* < 0.01).

**Table 2:** Birth weight, first-week body weight, average daily weight gain and rectal temperature of Awassi and Morkaraman lambs reared under cold climate conditions

Breed (Lamb)	Birth weight (kg)	BWFW (kg)	ADWG (kg)	Rectal temperature (°C)
Awassi (A)	4.15 ± 0.176	6.03 ± 0.149	0.250 ± 0.019	39.5 ± 0.1
Morkaraman (M)	4.65 ± 0.177	6.63 ± 0.211	0.276 ± 0.020	39.0 ± 0.1
p values	**0.053**	**0.031**	0.238	**< 0.01**

ADGW: Average daily weight gain, BWFW: Body weight in the first week, SEM: Standard error of means. Values are expressed as mean ± SEM

### Certain biochemical parameters in the serum of ewes and lambs

The results for certain serum biochemical parameters in Morkaraman and Awassi sheep and lambs are summarized in Tables 3 and 4. According to the results, triglyceride and GGT levels were significantly higher in Morkaraman sheep (*p* < 0.05). No statistically significant differences were observed between breeds for other serum biochemical parameters. In addition, glucose and BHBA concentrations differed significantly in lambs. Particularly, BHBA and glucose levels were higher in Awassi lambs compared to Morkaraman lambs (*p* < 0.05).

**Table 3:** Serum biochemical parameters of Awassi and Morkaraman ewes under cold climate conditions

	Strains		*P*
	Awassi (A)	Morkaraman (M)	
BHBA (ug/ml)	9.96 ± 0.683	9.16 ± 0.919	0.491
BUN (µmol/l)	106 ± 8.88	120 ± 6.96	0.231
NEFA (nmol/ml)	56.2 ± 1.95	61.4 ± 2.21	0.098
GGT (U/l)	43.2 ± 1.49	51.4 ± 2.28	**0.011**
TP (g/dl)	6.11 ± 0.96	5.90 ± 0.190	0.346
Alb (g/dl)	3.04 ± 0.058	3.09 ± 0.120	0.729
Glob (g/dl)	3.07 ± 0.096	2.81 ± 0.119	0.113
Glu (mg/dl)	63.3 ± 4.16	73.5 ± 3.60	0.082
TG (mg/dl)	17.8 ± 1.54	24.0 ± 0.548	**0.009**
Chol (mg/dl)	55.5 ± 2.93	52.4 ± 3.70	0.529
HDL (mg/dl)	33.8 ± 2.43	31.0 ± 2.53	0.440
LDL (mg/dl)	14.7 ± 1.23	16.7 ± 0.966	0.230

Alb: Albumin, BHBA: Beta-hydroxybutyric acid, BUN: Blood urea nitrogen, Chol: Cholesterol, GGT: Gamma-glutamyl transferase, Glu: Glucose, HDL: High-density lipoprotein, LDL: Low-density lipoprotein, NEFA: Non-esterified fatty acid, TG: Triglyceride, TP: Total protein, SEM: Standard error of means. Values are expressed as mean ± SEM

**Table 4:** Biochemical findings in blood serum of lambs

	Strains		*P*
	Awassi (A)	Morkaraman (M)	
BHB (ug/ml)	12.1 ± 1.21	9.23 ± 0.69	**0.043**
GGT (U/l)	1049 ± 110	1273 ± 93	0.146
TP (g/dl)	7.09 ± 0.223	6.91 ± 0.788	0.636
Alb (g/dl)	2.51 ± 0.046	2.44 ± 0.032	0.199
Glob (g/dl)	4.57 ± 0.258	4.48 ± 0.299	0.811
Glu (mg/dl)	115 ± 2.51	99 ± 4.14	**0.008**
IgG (ug/ml)	7.58 ± 0.619	9.28 ± 1.02	0.173

Alb: Albumin, BHB: Beta-hydroxybutyric acid, GGT: Gamma-glutamyl transferase, Glu: Glucose, TP: Total protein, SEM: Standard error of means. Values are expressed as mean ± SEM

### Colostrum quality

**Fig. 2:**
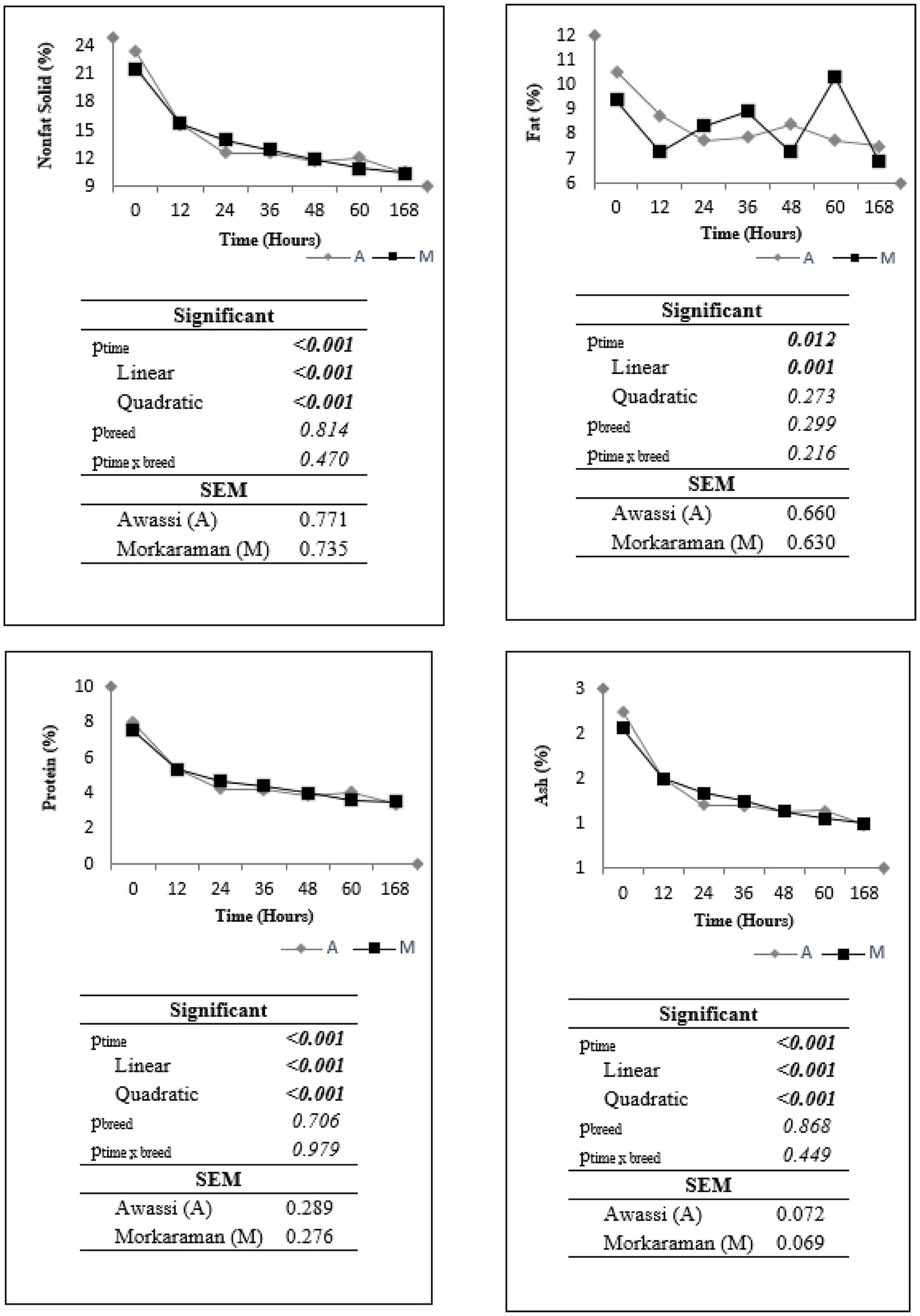
Changes in colostrum composition in sheep exposured to cold conditions in terms of time and breed. A: Awassi, M: Morkaraman, SEM: Standard error of means

**Fig. 3:**
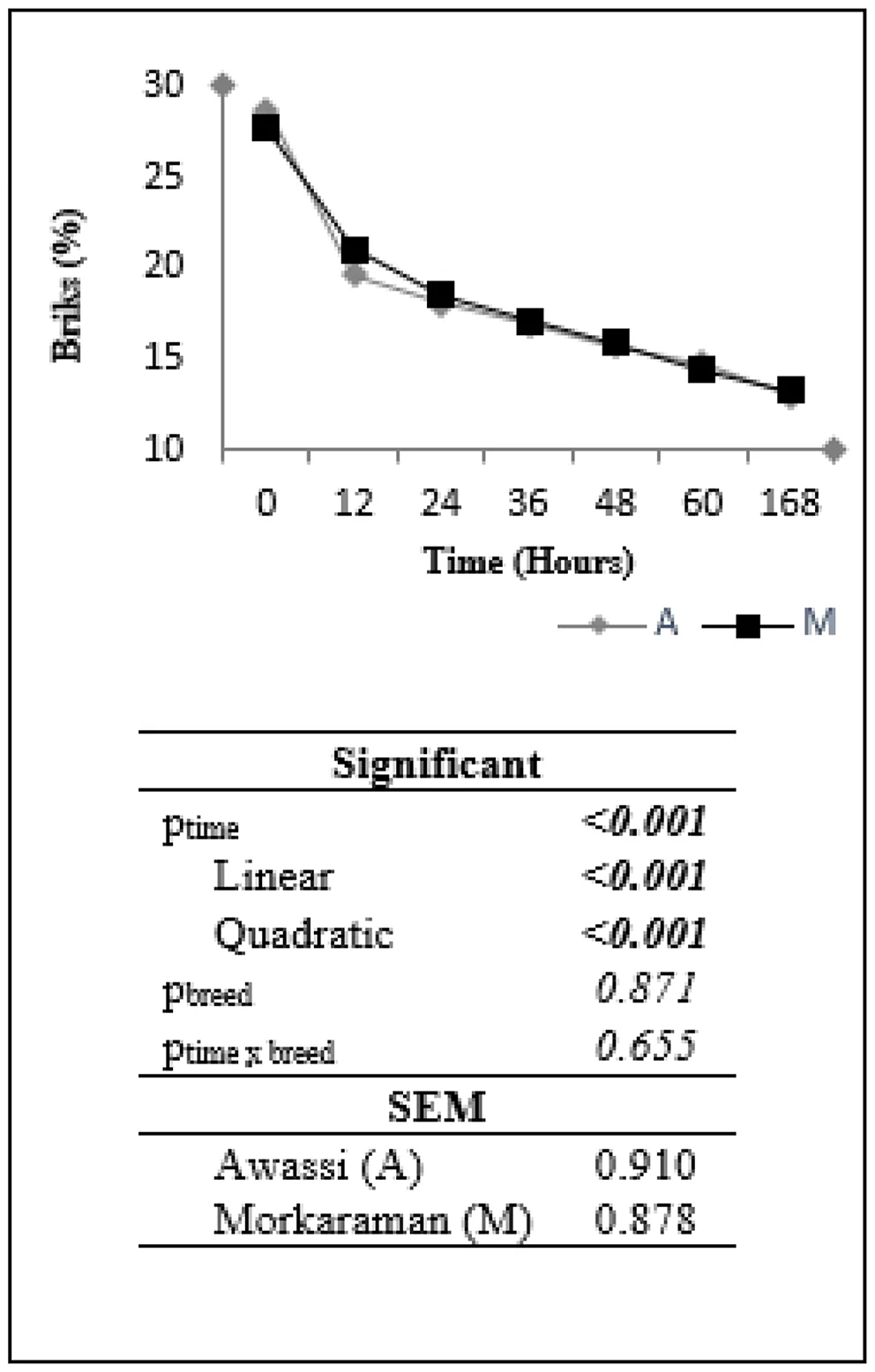
Brix values ​​of colostrum in sheep exposured to cold conditions in terms of time and breed. A: Awassi, M: Morkaraman, SEM: Standard error of means

**Fig. 4:**
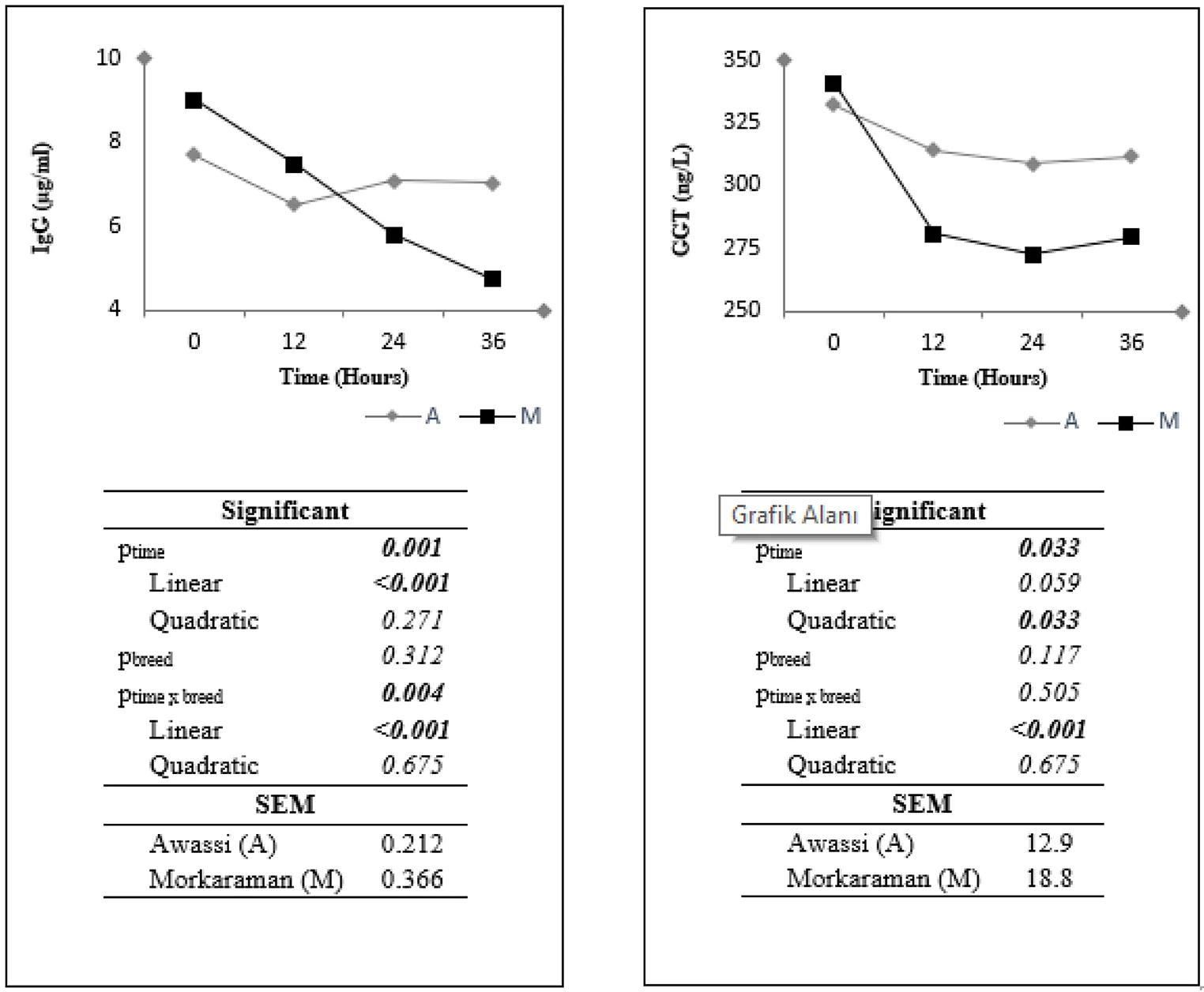
IgG and GGT levels in colostrum in sheep exposured to cold conditions in terms of time and breed. A: Awassi, M: Morkaraman, SEM: Standard error of means

Changes in colostrum composition in sheep exposed to cold conditions, in terms of time and breed, are shown in Figs. 2, 3 and 4. The findings showed that the non-fat solids, fat, protein, ash, Brix percentages, IgG, and GGT levels in colostrum decreased significantly from the first milking to the 7th day postpartum (*p* < 0.001). In addition, colostrum IgG and GGT concentrations were significantly affected by sheep breed (*p* < 0.01).

### Correlations between colostrum quality and serum biochemical parameters in ewes and lambs

The correlation between certain parameters in lambs and sheep, regardless of breed, and specific serum biochemical parameters is presented in Table 5. The findings revealed numerous significant correlations between serum biochemical parameters and colostrum quality. Colostrum Brix value and serum BHB level (r²=-0.491; *p* < 0.05) and colostrum GGT level (r²=-0.453; *p* < 0.05) were determined to be negatively correlated in ewe. Furthermore, colostrum GGT and colostrum IgG levels were found to be positively correlated (r^2^ = 0.504; *p* < 0.05) in cold condition. In both ewe (r^2^ = 0.516; *p* < 0.05) and lamb (r^2^ = 0.683; *p* < 0.01) serum, there was a significant correlation between the BUN levels and GGT levels. Additionally, there were significant correlations between the GGT (r²=0.482; *p* < 0.05) and TP (r²=-0.563; *p* < 0.01) levels in lamb serum and the GGT levels in ewe serum. Lastly, there was a significant positive correlation between the levels of albumin and total protein in ewe serum (r^2^ = 0.605; *p* < 0.01).

**Table 5:** Correlations between colostrum quality and some serum biochemical findings of ewes and lambs

					Ewe					Lamb	
		Brix (Colostrum) (%)	IgG (Colostrum) (ug/ml)	GGT (Colostrum) (ng/l)	BHBA (Serum) (ug/ml)	BUN (Serum) (µmol/l)	GGT (Serum) (U/l)	TP (Serum) (g/dl)	Alb (Serum) (g/dl)	GGT (Serum) (U/l)	TP (Serum) (g/dl)
	Brix (Colostrum) (%)	-	-0.244	-0.453^*^	-0.491^*^	-0.149	-0.382	-0.177	-0.078	0.047	0.337
	IgG (Colostrum) (ug/ml)	-0.244	-	0.504^*^	0.432	-0.345	-0.277	-0.226	-0.016	-0.263	0.401
	GGT (Colostrum) (ng/l)	-0.453^*^	0.504^*^	-	0.497^*^	-0.285	-0.233	0.178	0.11	-0.256	-0.185
Ewe	BHBA (Serum) (ug/ml)	-0.491^*^	0.432	0.497^*^	-	0.227	0.307	0.202	0.311	0.25	-0.18
	BUN (Serum) (µmol/l)	-0.149	-0.345	-0.285	0.227	-	0.516^*^	0.029	0.057	0.683^**^	-0.169
	GGT (Serum) (U/l)	-0.382	-0.277	-0.233	0.307	0.516^*^	-	0.255	0.314	0.482^*^	-0.563**
	TP (Serum) (g/dl)	-0.177	-0.226	0.178	0.202	0.029	0.255	-	0.605^**^	0.014	-0.297
	Alb (Serum) (g/dl)	-0.08	-0.02	0.11	0.31	0.06	0.31	0.605^**^	-	0.26	0.05
Lamb	GGT (Serum) (U/l)	0.047	-0.263	-0.256	0.25	0.683^**^	0.482^*^	0.014	0.259	-	-0.032
	TP (Serum) (g/dl)	0.337	0.401	-0.185	-0.175	-0.169	-0.563^**^	-0.297	0.049	-0.032	-

Alb: Albumin, BHB: Beta hydroxy butyric acid, BUN: Blood urea nitrogen, GGT: Gamma glutamyl transferase, TP: Total protein

## Discussion

Due to the demands of fetal growth, ewes frequently reduce their feed intake in the last few weeks of pregnancy, which can lead to an energy deficit (McGovern et al. 2015). This issue is especially noticeable in cold climates, where the energy deficit can seriously compromise the health of both lambs and ewes. Maternal energy deficiencies may also affect the quality of colostrum that lambs consume during postpartum. The present study aimed to determine how the metabolic profile of ewes, the health and neonatal performance of lambs, and the quality of colostrum in Awassi and Morkaraman breeds were impacted by cold-related conditions during pregnancy. Despite being fed the same diet, there were notable differences in the growth performance of Awassi and Morkaraman lambs that were exposed to cold. The mean birth weight of Awassi lambs was 4.15 kg, whereas Morkaraman lambs exhibited a higher mean value of 4.65 kg. By the end of the first week, the average body weight of Morkaraman lambs increased to 6.63 kg, slightly exceeding that of Awassi lambs (6.03 kg), indicating a modest advantage in early postnatal growth performance. Previous studies on lambs reared in cold cconditions has also reported breed-specific differences in birth weight, which lends support to the present findings (Dwyer and Margon 2016; Plush et al. 2016). The agreement of the current results with earlier research indicates that a newborn’s growth performance may be significantly influenced by their genetic background and ability to adapt to cold conditions. Morkaraman lambs’ increased birth weight may be due to the breed’s higher resistance to cold conditions. Because larger lambs tend to be more vigorous sucklers and more resilient to cold stress, a higher birth weight clearly benefits lamb performance (Dwyer et al. 2016).

The present study found that the rectal temperature of newborn Awassi lambs (39.5 ± 0.1 °C) was higher than that of newborn Morkaraman lambs (39.0 ± 0.1 °C). Lambs’ blood glucose levels and colostrum intake are directly related to their post-lambing rectal temperature. Furthermore, intaking colostrum after lambing raises the lambs’ metabolic rate and this increase in metabolic rate contributes to a rise in body temperature (Hamadeh et al. 2000). Breed can affect the physiological reaction to cold, according to a related study that looked at how lambs from four different sheep breeds responded to cold stress. The current study found that different breeds react differently to cold exposure in terms of their physiology and biochemistry (Plush et al. 2016). The agreement between these findings and earlier reports indicates that thermoregulatory responses to cold stress are strongly influenced by breed-specific metabolic and physiological traits.

Analyzing the metabolic profiles of ewes both before and after parturition is essential for determining the lambs’ health, milk production, and reproductive success (Hahem and El-Zarkouny 2017). In the present study, serum biochemical parameters were compared among ewes from different breeds to characterize breed-specific differences in metabolic and physiological status. According to the findings, Morkaraman ewes exhibited noticeably greater levels of triglyceride and GGT than Awassi ewes. The increased energy demands in the later stages of pregnancy may explain these differences. Additionally, the liver undergoes metabolic adaptations to meet these demands in ewes approaching parturition. Moreover, these metabolic alterations may have been influenced by the ewes’ exposure to cold environmental conditions. Other studies have reported similar results, indicating elevated triglyceride and GGT levels at lambing (Aly and Elshahawy 2016; Chagas et al. 2023; Temizel et al. 2018). The similarity of these results to previous studies supports the hypothesis that cold stress intensifies metabolic load during late gestation, particularly in breeds adapted to harsher climates.

Serum glucose and BHBA levels are important markers of energy metabolism in ruminant species (Hayırlı et al. 2012). In the present study, Awassi lambs showed greater levels of BHBA (12.1 ± 1.21 ug/ml) and blood glucose (115 ± 2.51 mg/dl) than Morkaraman lambs. For newborn lambs, colostrum serves as the primary energy source, because of its high nutritional content, colostrum is essential for thermoregulation (Agenbag et al. 2021). Awassi lambs’ elevated levels of BHBA and glucose may be related to birth weight, energy metabolism imbalances, and metabolic adaptations to cold stress. Interestingly, the average birth weight of Morkaraman lambs was higher than that of Awassi lambs. This difference may have influenced their metabolic profile during the early postnatal period. This discrepancy suggests that smaller lambs may rely more heavily on metabolic adaptations, such as increased glucose mobilization, to cope with cold stress. In addition, a study that investigated at hypothermia and hypoglycemia in newborn lambs found that healthy lambs had glucose levels of 103.2 ± 30.1 mg/dl, which consistent with the finding of the present study (Gök and Gül 2018). Likewise, a study on postnatal hypoglycemia in goat found that healthy kid had normoglycemic levels of 119.2 ± 6.3 mg/dl (Habibu et al. 2021). In consistent with findings from previous studies, the findings show that both lamb breeds in this study maintained glucose concentrations within the normoglycemic range. Although Morkaraman sheep are genetically adapted to harsh continental climates, under the environmental conditions in which the present study was conducted, Awassi sheep were found to exhibit a more effective physiological response to cold stress (Şahin and Kopuzlu 2022). The metabolic changes needed to adapt to the cold condition may be the cause of the higher serum glucose levels seen in Awassi lambs as compared to Morkaraman lambs (Doubek et al. 2003; Wang et al. 2019).

Colostrum composition and quality are influenced by several factors, including breed, parity, prepartum nutritional status, and the postpartum period (McGrath et al. 2016; Puppel et al. 2019). The present study found no breed-specific differences in the colostrum’s nonfat solids, fat, protein, ash, and Brix contents from Awassi and Morkaraman sheep while time had a significant effect on these parameters. This finding indicates that temporal changes after parturition may have a greater influence on colostrum composition than breed under cold climate conditions. The nutrient composition of colostrum, transition milk, and mature milk has been shown to decrease over time. This pattern has also been reported in Santa Inês sheep (Alves et al. 2015). Another study that investigated at how colostrum Brix values changed over time for a variety of sheep breeds, including Tuj, Awassi, Morkaraman, and Akkaraman, also found that Brix values decreased over time (Uysal et al. 2024).

In present study, it was found that Morkaraman sheep’s colostrum IgG and GGT concentrations decreased more quickly over time. When assessing the effectiveness of passive immunity transfer in lambs, these factors are crucial that GGT and IgG levels in colostrum are crucial biomarkers for estimating the likelihood of passive transfer failure (Belkasmi et al. 2022). The time-dependent decline in these parameters underscores the importance of ensuring that newborns ingest colostrum as soon as possible after lambing. The GGT activity has been identified as a reliable indicator of colostrum quality (Aydogdu and Guzelbektes 2018), and previous studies have consistently reported elevated GGT activity in colostrum (Rocha et al. 2012; Zarrilli et al. 2003). Colostrum GGT activity, colostrum IgG, and BHBA levels in ewes were found to positively correlate in the present study. On the other hand, colostrum Brix values and colostrum GGT activity were found to be negatively correlated. Interestingly, the positive relationship between colostrum GGT activity and IgG levels is consistent with previous a study (Viola et al. 2022). However, there is contradictory data because one study revealed no relationship between colostral GGT and IgG levels (Kaçar et al. 2021). These inconsistencies across studies may be attributed to differences in breed, management conditions, or sampling time. More study is necessary to understand the relationship among colostrum GGT activity, IgG concentration, and the metabolic profile of ewes, especially BHBA levels, in order to explain differences in previous research.

Urea is essential for nitrogen recycling and protein metabolism in ruminant animals. Urea has a high rate of circulation between the bloodstream and different tissues because of its small molecular size. The kind of protein in the feed that ruminants eat has a significant impact on their BUN levels (Tur et al. 2017). The present study found that the levels of lamb GGT and ewe BUN were positively correlated. Furthermore, there was a positive correlation between ewe serum GGT levels and lamb GGT and total protein levels. Lamb albumin levels and ewe total protein concentrations were also found to be positively correlated. A study indicates that GGT activity is frequently connected to protein synthesis and metabolism (Alves et al. 2015). Additionally, it has been demonstrated that ruminants’ GGT levels increase as they consume more colostrum after birth (Rocha et al. 2012). These findings suggest that the correlation between ewe BUN levels and lamb GGT levels might represent underlying dynamics of protein metabolism. Additionally, the positive relationship between lamb albumin concentrations and ewe total protein levels probably illustrates how the nutritional status of the ewe affects the health of the lamb (Ahmadzadeh et al. 2020).

Consequently, the present study show that the Morkaraman breed performed better in cold climates and that lambs born to ewes subjected to prolonged cold stress had higher birth weights than Awassi lambs. This finding indicates that Morkaraman lambs are better adapted to cold conditions in terms of birth weight and neonatal performance. Furthermore, ewes’ serum biochemical parameters were significantly impacted by prolonged cold exposure; Morkaraman ewes showed higher levels of triglycerides and GGT. The Awassi lambs’ exposure to cold stress was probably the cause of their elevated blood glucose and BHBA levels. Colostrum quality gradually declined over time, but Morkaraman ewes’ IgG and GGT concentrations decreased more quickly. Although Morkaraman sheep appear to be more resilient to cold stress, the rapid decline in colostrum IgG and GGT highlights the need for careful colostrum management in this breed to ensure adequate passive immunity transfer to lambs. These findings indicate breed-specific variations in how ewes and lambs react to cold stress and the effects of cold environments on both ewe metabolism and lamb development. This study has some limitations, including a relatively small sample size, restriction to a single geographic region, and the absence of genetic or hormonal analyses. These findings about these sheep breeds’ resistance to cold would be more broadly applicable in the future if larger sample sizes and a range of climatic conditions were used. In addition, the present results may have implications for breeding programs aimed at improving cold tolerance, as well as for management practices focused on nutritional support and neonatal care under cold environmental conditions. A more thorough understanding of these breeds’ mechanisms for cold tolerance would also be possible by carrying out genetic and metabolic analyses to investigate how they have adapted to cold climates.

## Data Availability

The raw data that support the research is available upon request to the corresponding author.
